# Equity and access to a regional child and youth mental health and addiction navigation service: a secondary analysis study

**DOI:** 10.1186/s12889-026-28043-3

**Published:** 2026-06-06

**Authors:** Scott J. Robson, Paula Cloutier, Ashley Radomski, Christine Polihronis, Kayla Beaudin, Josée Blackburn, Cindy Dawson, Mario Cappelli

**Affiliations:** 1 CHEO Research Institute c/o RI Administration Offices, 401 Smyth Road, Research Building 2, 2nd floor, Room 2119, Ottawa, ON K1H 8L1 Canada; 2 Knowledge Institute on Child and Youth Mental Health and Addictions, 695 Industrial Ave, Ottawa, ON K1G 0Z1 Canada; 3 CHEO, 401 Smyth Road, Ottawa, ON K1H 8L1 Canada; 4 Kid’s Come First Health Team, 401 Smyth Road, Ottawa, ON K1H 8L1 Canada; 5 1Call1Click.ca, 401 Smyth Road, Ottawa, ON K1H 8L1 Canada

**Keywords:** Adolescent, Child, Coordinated access, Equity, Infant, Mental health, Addiction, Substance use, Referral and consultation

## Abstract

**Background:**

To assess equity of access to a coordinated access service (1Call1Click.ca) in Eastern Ontario that connects infants, children, and youth to mental health, addictions and substance use health (MHASUH) support/services. Focusing on Social Determinants of Health (SDH), we analyze clientele for acuity and compare clientele demographics to service area population using census data.

**Method:**

Secondary analysis study of all clients (*N* = 9,694) (birth-21 years) who completed a 1Call1Click.ca intake between June 2021 and December 2023. We compared demographic characteristics (racial background, family income, and gender identity) of clients to regional data from the 2021 Census of Canada. We also examined whether SDH characteristics were associated with MHASUH need severity using standardized assessment tools (CASH Bundle).

**Results:**

Compared to the regional population, 1Call1Click.ca was accessed at higher rates by service clients who were: (1) cisgender-female, non-binary, and transgender individuals, (2) clients from families with the lowest and highest incomes, and (3) who identified with multiple and “other” racial backgrounds. Underrepresented groups included cisgender-males and moderate-income households ($60,000–89,999 CAD). Filipino, Japanese, Chinese, South Asian, and Indigenous identifying individuals accessed services at lower-than-population rates. Small differences in MHASUH acuity were observed for racial background, gender identity, and family income, though not uniformly across measures.

**Conclusions:**

Clients from historically disadvantaged groups accessed 1Call1Click.ca at rates largely representative of or exceeding those in the regional population. Differences in acuity were small and inconsistently identified. These findings suggest potential for coordinated access services in increasing equity for children and youth historically disadvantaged by SDH.

**Supplementary Information:**

The online version contains supplementary material available at 10.1186/s12889-026-28043-3.

## Background

Canadians identify accessibility and equity as top challenges affecting healthcare [1]. Nearly half of people reporting mental health challenges in their lifetime experience the onset of those challenges by the age of 18 [2]. However, many do not access care within a year of symptom onset and estimates suggest that 200,000 children in Ontario with serious mental health, addictions, and substance use health (MHASUH) needs have no contact with services to treat those challenges [3, 4]. This is a challenge that needs to be addressed, as delays in treatment access are associated with greater and longer-term challenges [5–9]. Of the many barriers to accessing MHASUH care, “not knowing where to go” is often cited as the greatest, with systemic barriers such as discrimination and expense contributing to the problem [10, 11]. Investing in removing the barriers to accessing infant, child and youth MHASUH care is an important step towards early intervention.

### Barriers to child and youth mental health access

Social Determinants of Health (SDH) are nonmedical social factors that influence health outcomes over the lifespan [12]. The interaction between the social and cultural structures of a place and SDH characteristics produce systemic inequities that can impact both the health of an individual and their ability to access healthcare. While we refer to SDH related characteristics (gender identity, racial background, family income) throughout the manuscript, it is because these are the characteristics of individuals that are measured in the data we analyze, not because of a causal relationship to health outcomes. That is to say, the effects of SDH on one’s health are not inherent to possessing a particular feature (for instance, a specific racial background or gender identity) but rather the way the people and systems amongst which a person is embedded respond to them. SDH are the genuine effects on health that benefit some and disadvantage others through secondary effects related to systemic and interpersonal discrimination (such as racism or sexism) or inequities resulting from wealth disparity.

Systemic SDH-related barriers including language, access to primary care, income inequality, or discrimination based on racial background, gender, or sexual orientation, can delay or prevent access to care [13–21]. Barriers faced by historically disadvantaged groups can be of the same type as those faced by others (e.g., determining where to go), the same type but of a greater scale to overcome (e.g., cost, distance, internet access), and can include additional challenges not faced by others (e.g., discrimination). In addition, others have noted effects of disparities in MHASUH prevalence and acuity in relation to factors like income inequality [22–24], gender identity [25, 26], and racial background [27]. Measuring MHASUH needs along with SDH factors are important steps toward increasing access and equity [28].

### Data, SDH, and equity of access

Without understanding the patterns of access to care prior to the implementation of an intervention, it is difficult to be precise about the impact of that intervention. Unfortunately, SDH data elements have historically gone uncollected at the client level. Measuring MHASUH need, as well as sociocultural and SDH factors including age, racial background, income level, and gender are important steps in increasing access and equity. This data collection can enable the observation of trends in service utilization and provide guidance in developing early intervention strategies along with more effective or tailored care and services [28].

1Call1Click.ca is a regional coordinated access and navigation service covering parts of Eastern Ontario that aims to address navigation barriers by connecting infants, children, youth and families directly to MHASUH care matching their needs. 1Call1Click.ca has the explicit goal of increasing access to care and employs a data-driven approach to identify and address access disparities.

Intake workers at 1Call1Click.ca are knowledgeable about publicly funded local MHASUH resources (such as community-based youth mental health agencies or specialized hospital programs), including the scope of service and admission requirements for those organizations. The intake workers ask demographic questions, discuss client needs and goals, and use the CASH Bundle Screening Tools (including the CRAFFT 2.1 + N, Ask Suicide Questions (ASQ), and HEADS-ED (Over and Under 6) to assign clients a *Level of Need* based on *Ontario’s Roadmap to Wellness* [29–37]. They directly connect each client to one or more MHASUH resources, making an appointment for the client and sharing the intake information with the referred resource.

Given the lack of baseline data, and the complex interactions between SDH, barriers to care, prevalence, and acuity of need, it is difficult to set a clear expectation for the distribution of clients seeking MHASUH care. For the purposes of this work, we expect that if the service is meeting its goal of facilitating access to clients from groups who have traditionally faced disproportionate barriers to accessing care, that clients from those groups would appear at the service levels either roughly equal to or exceeding population proportions of those demographics within the service area.

### Study objectives

To examine representativeness of access patterns through a SDH lens, we compared the proportions of the SDH characteristics of gender identity, racial background and family income of those accessing 1Call1Click.ca to regional population proportions reported in the publicly available 2021 Canada Census data [38–43]. We examined MHASUH acuity through screening tool outcomes and assigned *Level of Need* in relation to each of gender identity, racial background and family income to determine if there were differences in acuity related to these SDH factors.

## Methods

### Study design

We conducted two sub-studies based on this cohort:

1. We compared the proportions of the SDH characteristics gender identity, racial background and family income of those accessing 1Call1Click.ca to regional population proportions using publicly available 2021 regional Census data.
2. Within the cohort, we examined differences in MHASUH acuity, as captured by clients’ assigned *Level of Need* and screening tool outcomes, on the basis of the SDH characteristics of gender identity, racial background and family income.

This study was approved by the CHEO Research Ethics Board (#22/27X). This study follows the Strengthening the Reporting of Observational Studies in Epidemiology (STROBE) guideline [44].

### Participants and inclusion criteria

We used a secondary analysis study design examining all clients who had an intake with 1Call1Click.ca between June 1, 2021, and December 31, 2023 (*n* = 9694). Clients ranged in age from birth to 21 years (Table 1). Not all measures are applicable to all clients and SDH related demographic questions at 1Call1Click.ca are optional, therefore pairwise deletion was used to limit specific analyses to include only clients with an associated data point.

**Table 1:** Demographics of 1Call1Click.ca and corresponding Census data

	1Call1Click.ca Demographic Group	Number of Clients	Percentage of Clients		
Age	0 to 2 years	84	0.9%		
	3 to 5 years	628	6.5%		
	6 to 8 years	1149	11.9%		
	9 to 11 years	1639	16.9%		
	12 to 14 years	2827	29.2%		
	15 to 17 years	3152	32.5%		
	18 to 21 years	215	2.2%		
Level of Need	General Population *(Population Based Health Promotion and Prevention)*	100	1.0%		
	Low Need *(Early Intervention and Treatment)*	1982	20.4%		
	Moderate Need *(Targeted to Moderate Mental Health*,* Addictions*,* and Substance Use Health Needs)*	5361	55.3%		
	Moderate to Severe Needs *(Intensive and Specialized Care)*	1478	15.2%		
	Severe or Complex Needs *(Highly Specialized and Intensive Care)*	61	0.6%		
	No Data	712	7.3%		

	1Call1Click.ca Demographic Group	Number of Clients	Percentage of Clients	Percentages for Analyses	Census Percentages
Gender Identity	Cisgender female	5275	54.5%	56.4%	50.8%
	Cisgender male	3808	39.6%	41.0%	48.7%
	Non-Binary	136	1.4%	1.4%	0.2%
	Transgender	151	1.6%	1.6%	0.2%
	*Trans Male*	123	1.3%		
	*Trans Female*	28	0.3%		
	Other	50	0.5%		
	Agender	< 10	< 0.1%		
	Two Spirit	< 10	< 0.1%		
	Prefer not to answer/ No response	265	2.7%		
Racial Background	Arab	293	3.0%	3.2%	4.0%
	Black	372	3.8%	4.1%	6.0%
	Chinese	101	1.0%	1.1%	3.2%
	Filipino	17	0.2%	0.2%	1.1%
	Indigenous	188	1.9%	2.0%	3.4%
	Japanese	< 10	< 0.1%	0.1%	0.2%
	Korean	20	0.2%	0.2%	0.3%
	Latin American	98	1.0%	1.1%	1.1%
	Prefer not to answer/ No response	515	5.3%		
	Other	118	1.2%	1.3%	0.3%
	South Asian	164	1.7%	1.8%	4.3%
	Southeast Asian	97	1.0%	1.1%	1.1%
	West Asian	54	0.6%	0.6%	0.9%
	White	6931	71.5%	75.5%	73.4%
	Multiracial	721	7.4%	7.9%	0.8%
	* Indigenous/White*	* 165*	* 1.7%*		
	* Black/White*	* 168*	* 1.7%*		
	* Latin American/White*	* 59*	* 0.6%*		
	* Chinese/White*	* 44*	* 0.5%*		
	* Arab/White*	* 40*	* 0.4%*		
	* South Asian/White*	* 38*	* 0.4%*		
	* Southeast Asian/White*	* 31*	* 0.3%*		
	* White/Other*	* 24*	* 0.2%*		
	* Filipino/White*	* 17*	* 0.2%*		
	* Japanese/White*	* 17*	* 0.2%*		
	* West Asian/White*	* 13*	* 0.1%*		
	* Other multiracial*	* 104*	* 1.1%*		
Family Income (CAD)	$0 to $29,999	545	5.6%	14.1%	10.1%
	$30,000 to $59,999	651	6.7%	16.9%	18.0%
	$60,000 to $89,999	540	5.6%	14.0%	19.0%
	$90,000 to $119,999	602	6.2%	15.6%	15.8%
	$120,000 to $149,999	438	4.5%	11.3%	12.1%
	$150,000 or higher	1084	11.2%	28.1%	25.0%
	Do not know	4022	41.5%		
	Prefer not to answer/ No response	1812	18.7%		

Demographic questions at 1Call1Click.ca include response options not available on the census. In cases where identities not reported on the census were endorsed, or where clients selected the options “Prefer not to answer” or “I do not know”, we treated these as missing data for the purpose of population level comparisons. Numbers of clients endorsing each characteristic and who were assessed with each screening tool can be found in Appendix A.

### Setting

1Call1Click.ca serves clients within an area of Eastern Ontario home to 1,513,920 individuals per the 2021 census [38–43]. The service area includes a large city of over 1 million people, 3 smaller cities (25,000–50,000) and a rural area including many smaller municipalities.

### Data collection

#### Demographic data

Data were collected by intake workers at 1Call1Click.ca who routinely ask about demographic characteristics including the clients’ self-identified racial backgrounds, gender identity, and family income.

#### Clinical information

The CASH (CRAFFT, ASQ, HEADS-ED) Screening Tool Bundle is used to screen clients for MHASUH challenges and acuity. The bundle includes the HEADS-ED [45, 46], used for children 6 years and older, and the HEADS-ED Under 6 [36, 47], which is used for all clients under 6. Total scores for each HEADS-ED tool range from 0 to 14, with higher scores indicating greater clinical acuity. The CASH Bundle also includes the CRAFFT 2.1 + N [30, 31, 48], which is used with clients aged 12 and older to screen for substance use, and the Ask Suicide Questions (ASQ), which screen for suicidality [32, 33].

Intake workers use the CASH Bundle to help determine the client’s *Level of Need* (Table 1), derived from a stepped care model aligned with Ontario’s Roadmap to Wellness [37]. There are five *Level of Need* categorizations, each an indicator of acuity and used to help the intake worker determine which MHASUH community or hospital-based service to connect each client to. Client responses are recorded in the Electronic Medical Record database (EPIC) during the intake.

#### Community demographics

Census data were gathered and published by Statistics Canada. The researchers gathered this information from the Statistics Canada website [38–43]. In instances where the census response options did not match those of the 1Call1Click.ca dataset, some adjustments were made for the purpose of comparison. These adjustments are explained in detail in Appendix B.

### Data analysis

Analyses were performed using JASP 0.19 [49]. Data from the 2021 Canadian census for the 1Call1Click.ca service region were used to establish the proportions of SDH characteristics including gender identity, family income, and racial background (Table 1) in the service area [38–43]. These proportions were compared against the proportions of clients endorsing that characteristic at 1Call1Click.ca using a chi-square goodness-of-fit test. To limit type one error rate, pairwise analyses were limited by ranking proportional residuals in descending order and performing binomial tests with a sequential Bonferroni-Holm post-hoc correction until the tests no longer reached significance. Odds ratios were used to measure effect size.

A chi-square test of independence was used to examine categorical differences in acuity (CRAFFT, ASQ, *Level of Need*) in relation to SDH characteristics, with Standardized Pearson Residuals used to identify specific differences. ANOVA were used to compare HEADS-ED total scores across SDH characteristics. Bonferroni adjusted p-values are presented for ANOVA post-hoc comparisons. Because of frequent missing responses (60.2%), a sensitivity analysis was conducted for family income data.

## Results

To examine regional representativeness, 1Call1Click.ca proportions of SDH characteristics are compared with proportions of those characteristics within the 1Call1Click.ca service area as reported by the census. We then examined how SDH variables were associated with acuity and *Level of Need* using the CASH bundle (Table 1).

### Gender

#### Representativeness

**Fig. 1:**
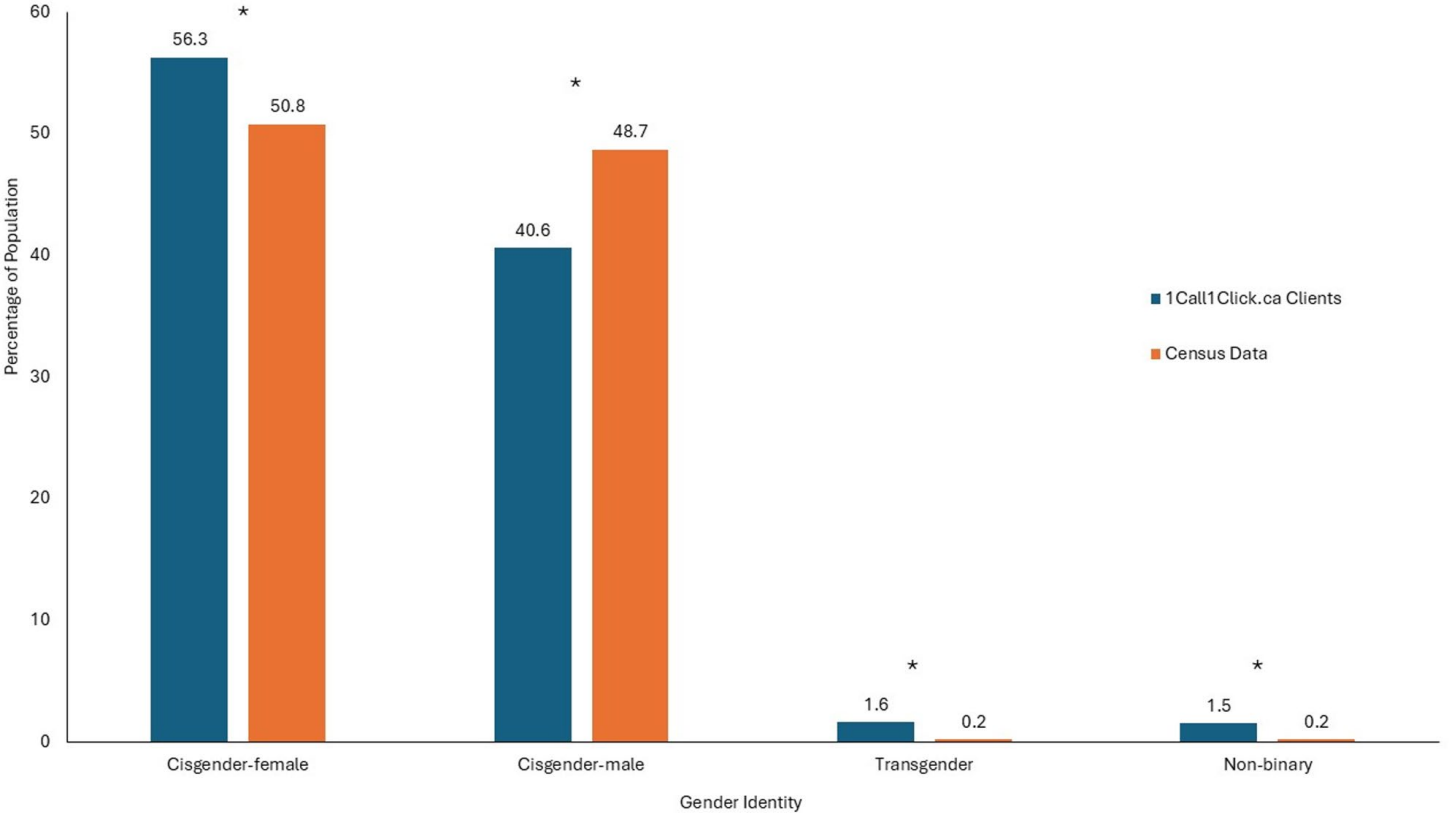
The gender identities of the clientele of 1Call1Click.ca as compared to the proportions of those identities in the regional population, as captured in the 2021 Census. * indicate comparisons that are significant at *p*<.05

We observed a statistically significant small-to-moderate difference in the gender distribution of clients at 1Call1Click.ca and the regional population (χ^2^[3, 9370]) = 1710.124; *p* < .001, Cramer’s V = 0.25). Non-binary and transgender clients were over-represented compared to the population frequencies with a large effect size (OR > 5) (Fig. 1) [50]. Cisgender-male clients were under-represented as compared to the regional population. The smallest effect observed was a statistically significant over-representation of cisgender-female clients relative to the regional population.

We made the post-hoc observation that the gender varied by age, with 58% of clients 10 and under were cisgender-male, while 61% of clients over 10 identified as cisgender-female. To determine if the observed effect could be explained by shifts in age patterns, we performed an unplanned Mantel-Haenszel chi-square test, splitting by age (10 or younger, over 10), to compare census proportions to observed client proportions. Due to small cell sizes in the 10 or younger group, non-binary and transgender clients were not included in this component of the analysis. We found that the effect of gender remained after controlling for age (χ^2^ [1]) = 91.107; *p* < .001).

#### CASH bundle

**Fig. 2:**
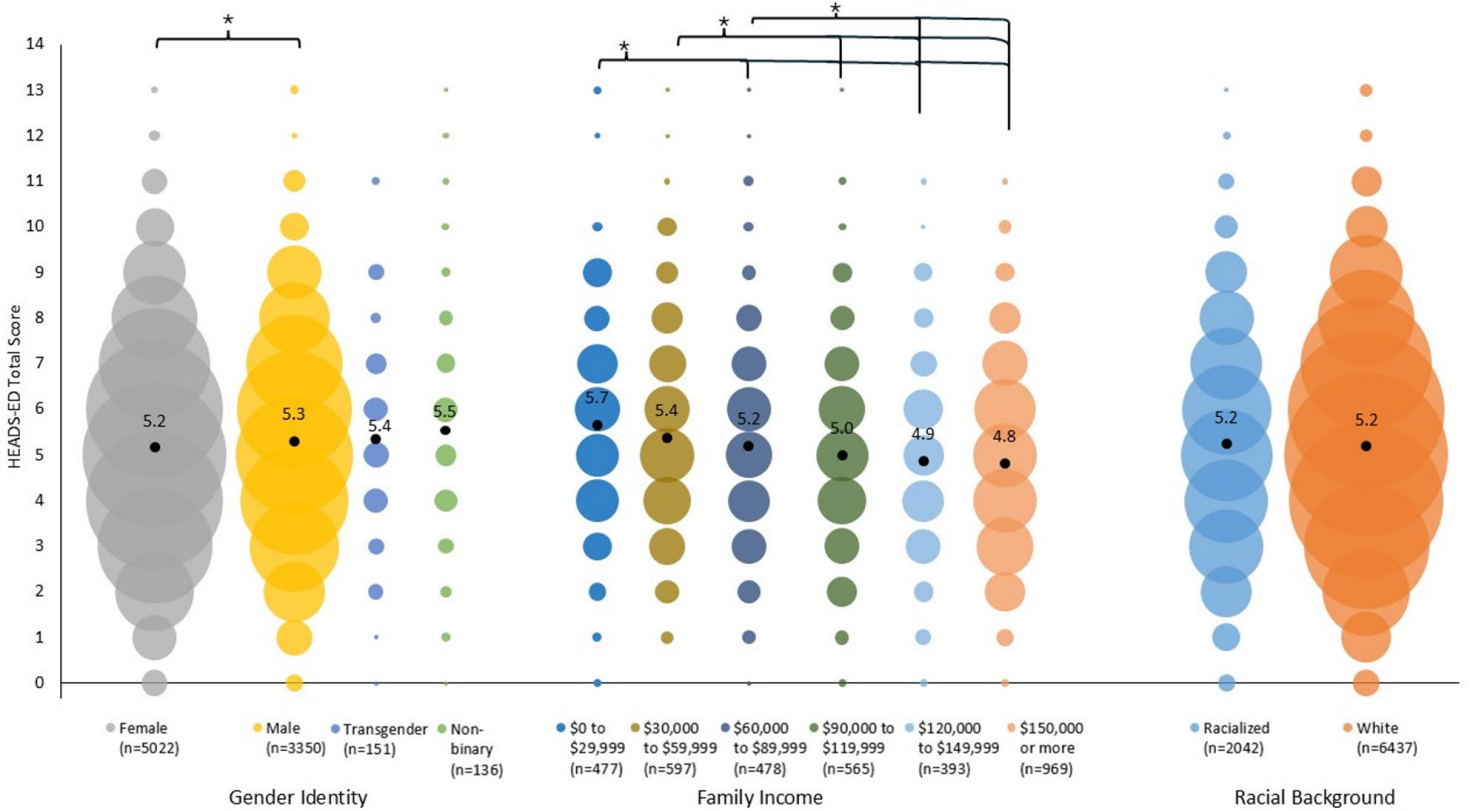
HEADS-ED total scores as distributed across the characteristics of gender, family income, and racial background. * indicate comparisons that are significant at *p*<.05

**Fig. 3:**
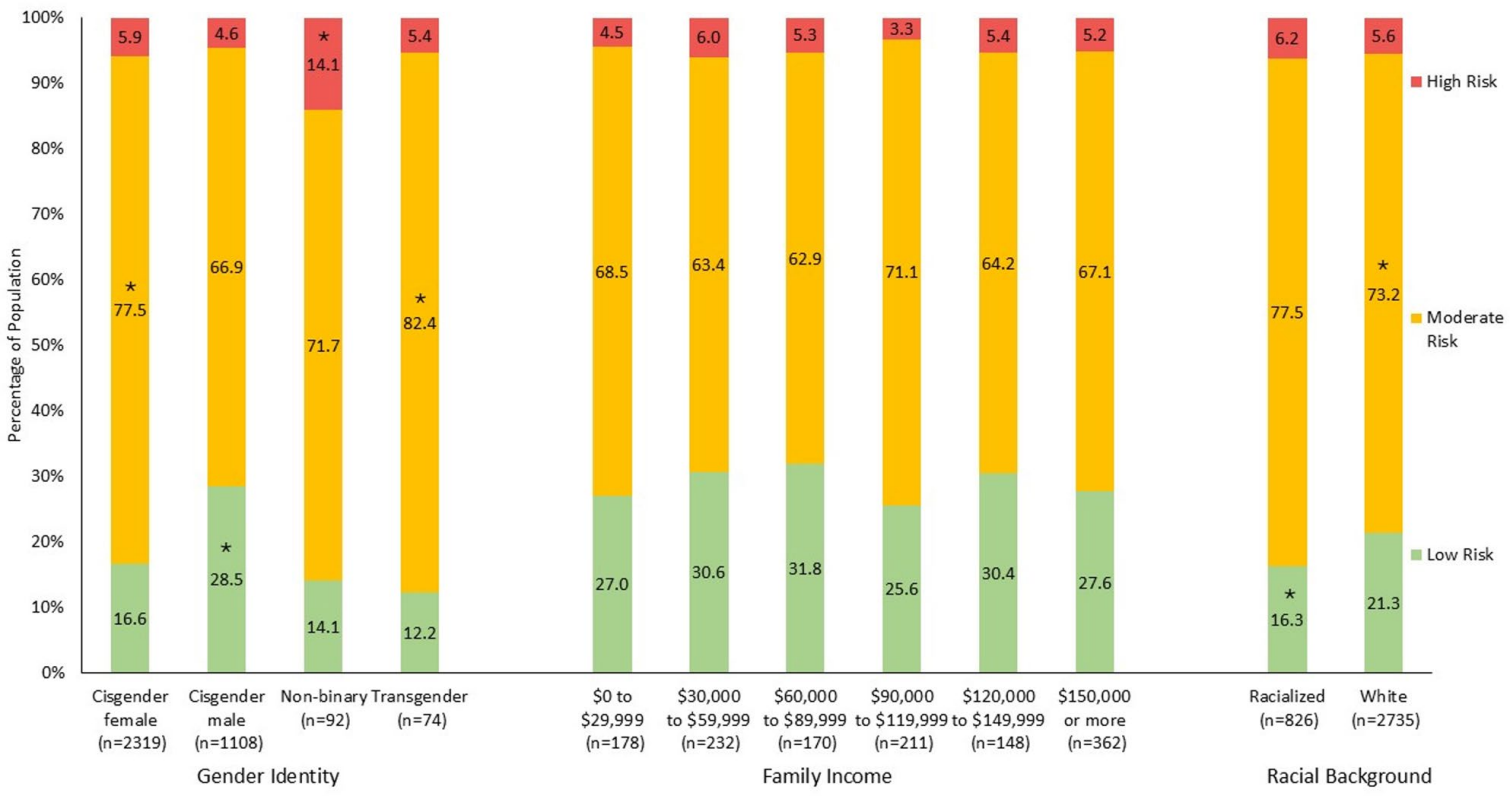
Risk outcomes as determined by the ASQ substance use screening tool, as distributed across gender identity, family income, and racial background. * indicates a significant difference indicated by a standardized Pearson Residual exceeding + 1/-1

Statistically significant gender differences in acuity were found on the HEADS-ED and ASQ tools. HEADS-ED total scores differed significantly (F(3,8655) = 4.235, *p*=.005, η^2^ = 0.001), with pairwise comparisons indicating a small effect (*t* = *2.897*, *p=*.023, *d =* 0.065) in which cisgender-male clients had higher HEADS-ED totals than cisgender-female clients (Fig. 2). Likewise, there was a statistically significant relationship between gender and suicide risk on the ASQ (χ^2^[6, 3593]) = 83.213 *p*<.001, Cramer’s V = 0.108) (Fig. 3). Standardized Pearson Residuals of more than + 1/-1 indicate that cisgender-female and transgender clients were over-represented in the non-acute positive screen (Moderate Risk) outcome and non-binary clients were present in higher-than-expected frequencies in the acute positive screen (High Risk) outcome. Cisgender-male clients were higher-than-expected in the negative screen (Low Risk) category of the ASQ.

**Fig. 4:**
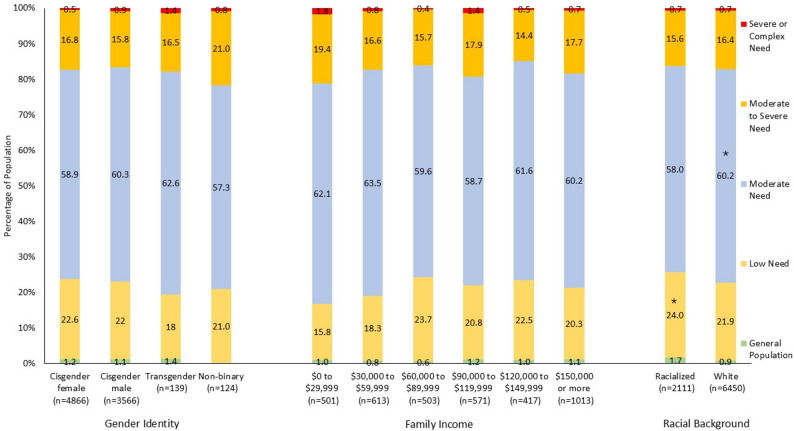
The proportions of clients at each Level of Need, as distributed across gender identity, family income, and racial background. * indicates a significant difference indicated by a standardized Pearson Residual exceeding + 1/-1

**Fig. 5:**
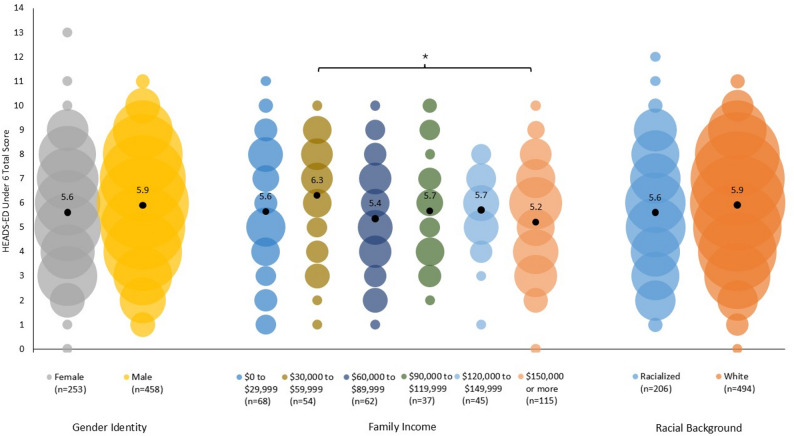
HEADS-ED Under 6 total scores as distributed across the characteristics of gender, family income, and racial background. * indicate comparisons that are significant at *p*<.05

**Fig. 6:**
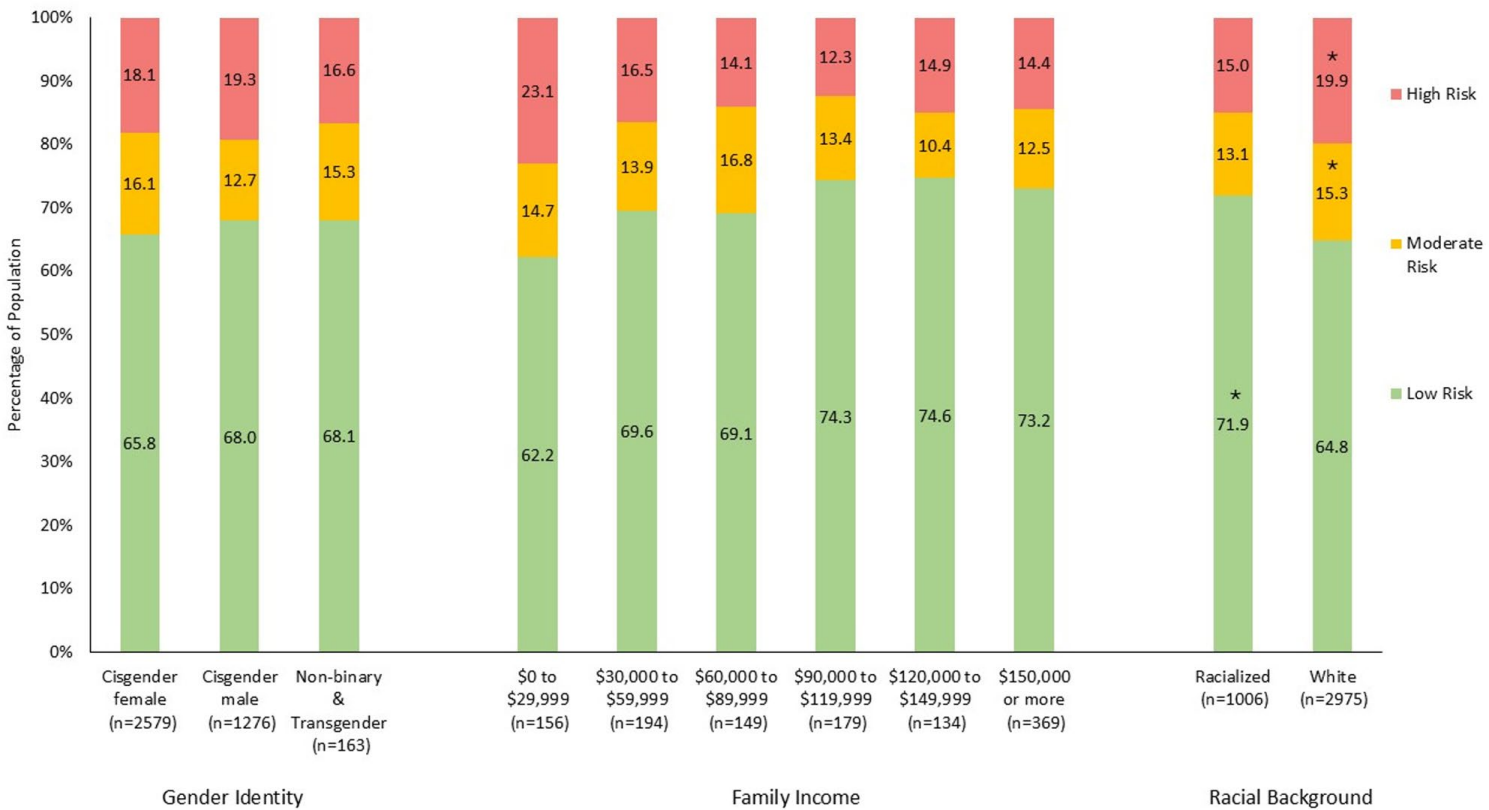
Risk outcomes as determined by the CRAFFT substance use screening tool, as distributed across gender identity, family income, and racial background. * indicates a significant difference indicated by a standardized Pearson Residual exceeding + 1/-1

There were no observed differences on the basis of gender identity detected for *Level of Need* (χ^2^[12, 8695]) = 11.630; *p=*.476, Cramer’s V = 0.021) (Fig. 4), HEADS-ED Under 6 total score (F(1, 709) = 3.339, *p*=.068) (Fig. 5), or CRAFFT 2.1 + N substance use risk outcomes (χ^2^[4, 4018]) = 8.156; *p=*.086, Cramer’s V = 0.032) (Fig. 6).

### Income

#### Representativeness

**Fig. 7:**
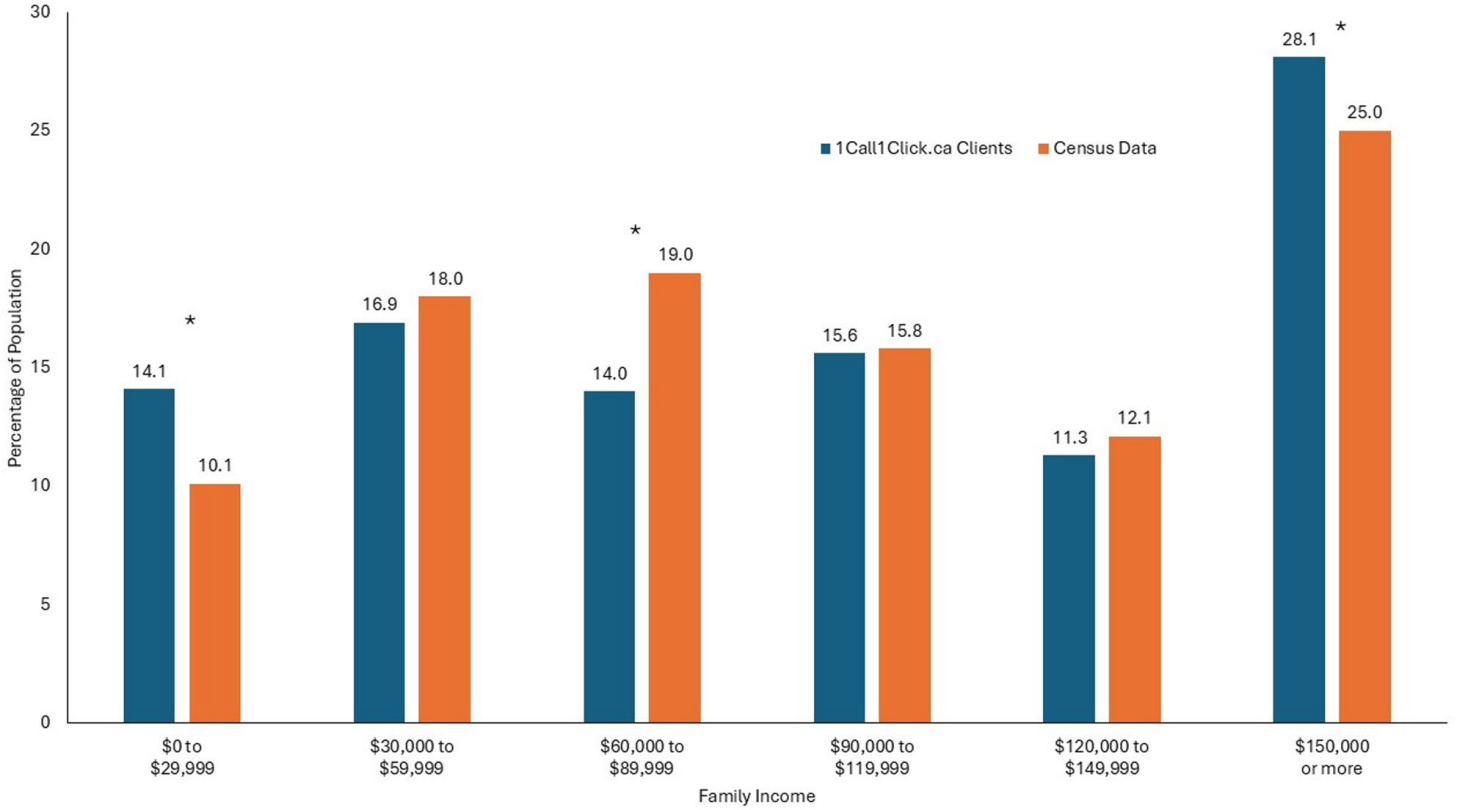
The family incomes of the clientele of 1Call1Click.ca as compared to the proportions of family income ranges in the regional population, as captured in the 2021 Census. * indicate comparisons that are significant at a *p*<.05

We observed a very small but statistically significant difference in family income distribution compared to the regional population levels (χ^2^[5, 3860]) = 131.888; *p* < .001, Cramer’s V = 0.09). Pairwise comparisons indicated that clients at the lowest income (*$0 to $29*,*999*) and highest income (*$150*,*000 or more)* exceeded the population proportions, while clients at the *$60*,*000 to $89*,*999* income level were under-represented and other income levels did not differ from population levels. (Fig. 7). Each of these differences indicates a small effect size (0.66 < *OR* < 1.5) [50].

#### CASH bundle

We observed statistically significant differences for family income level in HEADS-ED and HEADS-ED Under 6 total scores. There was a small but significant effect (F(5, 3473) = 15.817, *p*<.001, η^2^ = 0.022), revealing an inverse relationship wherein children and young people from lower income families are more likely to have higher HEADS-ED total scores and higher income families likely to have lower HEADS-ED total scores (Fig. 2).

Additionally, analyses revealed a small but significant effect (F(5, 375) = 2.274, *p*=.047, η^2^ = 0.029) for total scores on the HEADS-ED Under 6 across family income levels. Pairwise comparisons indicate a single statistically significant difference (*t* = 3.21, *p* = .022, *d* = 0.53) between the HEADS-ED Under 6 total scores of the second lowest ($30,000-$59,999) and the highest income group (*$150*,*000 or more*) (Fig. 5).

For family income, no statistically significant differences in acuity were observed for *Level of Need* (χ^2^[20, 3618]) = 26.185; *p* = .160, Cramer’s V = 0.04) (Fig. 4), the CRAFFT 2.1 + N (χ^2^[10, 1181]) = 12.795; *p =* .235, Cramer’s V = 0.074) (Fig. 6), or the ASQ (χ^2^[10, 1301]) = 5.538; *p =* .852, Cramer’s V = 0.046) (Fig. 3).

#### Sensitivity analysis

Because of the high proportion of clients who did not know or did not report their family income, we compared the CASH bundle outcomes and *Level of Need* between those who provided income data and those who did not, to test against possible limitations in the generalizability of our findings. Comparing the HEADS-ED total scores between those who provided income data (M = 5.12, SD = 2.0) and those who did not (M = 5.32, SD = 2.1), we found a small but statistically significant difference between the two groups (*t*(8968)=-4.519, *p*<.001, *d=*-0.1). A statistically significant difference (*t*(722)=-3.043, *p=*.002, *d=*-0.2) was observed between the HEADS-ED Under 6 total scores of those who provided income information (M = 5.6, SD = 2.1) and those who did not (M = 6.0, SD = 2.0). A chi-square test of independence was used to determine differences between those providing income data and those who did not along their assigned *Level of Need* found a very small but statistically significant effect as well (χ^2^[4, 8982]) = 19.069; *p<* .001, Cramer’s V = 0.046). A statistically significant but small difference was observed from a chi-square test of independence on CRAFFT risk level (χ^2^[2, 4159]) = 12.579; *p=*.002, Cramer’s V = 0.055) and the ASQ (χ^2^[4, 3744]) = 92.008; *p<* .001, Cramer’s V = 0.157).

### Racial background

#### Representativeness

**Fig. 8:**
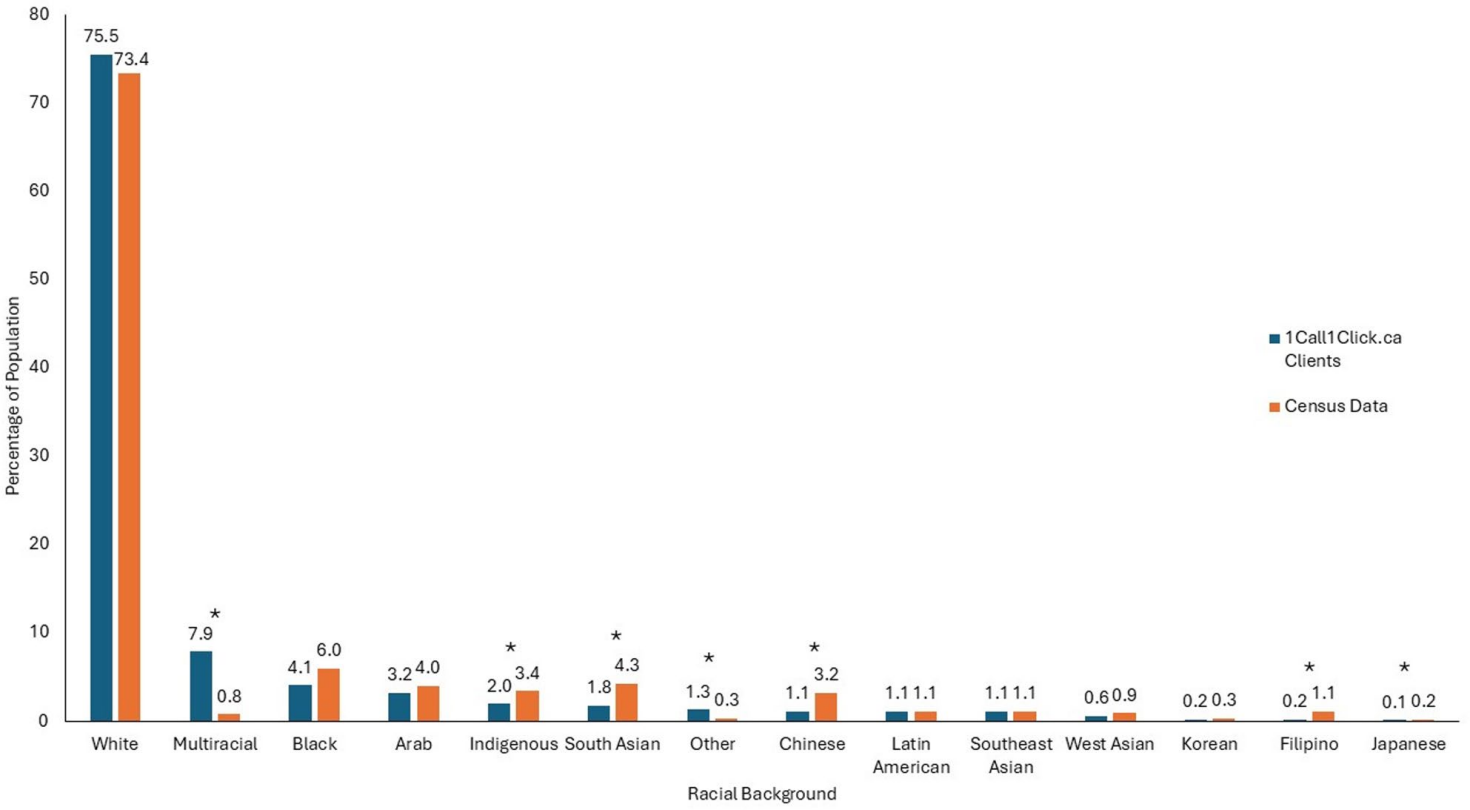
The racial backgrounds of the clientele of 1Call1Click.ca as compared to the proportions of those backgrounds in the regional population, as captured in the 2021 Census. * indicate comparisons that are significant at a *p*<.05

We observed a statistically significant effect of racial background endorsed by clients at 1Call1Click.ca compared to the regional population (χ^2^[13, 9179]) = 6655.433; *p* < .001, Cramer’s V = 0.236) (Fig. 8), indicating a small-to-medium effect. Multiracial clients were represented at 10 times the population level, where the “Other” background was selected at over 4 times the population level. Filipino, Japanese, Chinese, South Asian, and Indigenous populations are below census population levels. None of the other 7 racial backgrounds reported differed from census levels.

#### CASH bundle

There were significant differences observed for racial background on *Level of Need*, the CRAFFT, and ASQ. We observed a significant but very small effect of racial background on the *Level of Need* for clients at 1Call1Click.ca (χ^2^[4, 8561]) = 12.096; *p* = .017, Cramer’s V = 0.04). Standardized Pearson Residuals exceeding + 1/- 1 indicate there are significantly more racialized individuals in the General Population *Level of Need*, the lowest *Level of Need*, while there are more White individuals in the Moderate Need level (Fig. 4).

Analysis reveals a small but significant difference in CRAFFT 2.1 + N outcomes by racialization (χ^2^[2, 3981]) = 17.666; *p*<.001, Cramer’s V = 0.067) (Fig. 6). Standardized Pearson residuals greater than + 1/-1 indicate that White identifying individuals are present in the Moderate Risk and High-Risk outcomes of the CRAFFT 2.1 + N in higher-than-expected levels, while racialized clients are more frequent in the Low-Risk outcome. Likewise, we observed a very small but statistically significant difference in ASQ outcomes by racialization (χ^2^[2, 3561]) = 9.695; *p*=.008, Cramer’s V = 0.052) (Fig. 3). Pearson residuals indicate that more-than-expected White clients are in the negative screen (Low Risk) ASQ outcome while more racialized individuals are in the non-acute positive screen outcome (Moderate Risk).

No statistically significant effects of racial background were observed for HEADS-ED (F(1, 8477) = 0.933, *p*=.334) (Fig. 2) or HEADS-ED Under 6 total scores (F(1, 698) = 3.170, *p*=.075) (Fig. 5).

## Discussion

### Summary of findings

Individuals from historically disadvantaged groups are accessing 1Call1Click.ca in proportions approximate to or exceeding population levels, though some racial identities are present in lower-than-population frequencies. In our measures of acuity, we see either no differences based on SDH characteristics or differences with small effect sizes. There are no SDH that we examined for which a difference in acuity was detected by each of the screening tools used.

### Gender

A high proportion of cisgender-female clients was observed relative to cisgender-male clients. This may be similar to a previously observed effect where males are less likely to seek out mental health treatment compared to females due to the stigma associated with seeking help [3, 18, 51, 52]. Others have noted that early onset disorders are more often detected in boys, whereas adolescent onset disorders such as anxiety are more frequent in girls [53]. This age and gender related imbalance was observed in the 1Call1Click.ca dataset; 58% of clients 10 and under were cisgender-male, while 61% of clients over 10 identified as cisgender-female. This imbalance in age and gender in seeking MHASUH care deserves further study. Specifically, to if these imbalances in care seeking behaviour related to age are reflective of sex-related differences in symptom onset or of societal gender-related expectations of the behavioural and emotional states children and youth at different stages of their development.

Higher-than-population proportions of individuals who identified as transgender or non-binary were observed at 1Call1Click.ca. This may be the result of a higher incidence MHASUH challenges in LGBTQ2SIA+ youth, as has been described elsewhere [25, 54, 55]. It should be noted though, that the census data available reflect the percentage of the regional population (aged 15+) who identified as non-binary or transgender. The same report indicates the proportion of the population identifying as transgender or non-binary is higher among youth (the population eligible to engage with 1Call1Click.ca) and within urban populations, but does not provide specifics for youth at the community level [56–58]. However, though the reported proportions of Canadian youth (aged 15–19) who identify as transgender (0.44%) and non-binary (0.29%) are nearly double the proportion of the area population (0.24% and 0.19% respectively), that proportion remains substantially lower than the proportions of transgender (1.19%) and non-binary (1.45%) youth presenting to 1Call1Click.ca.

### Racial background

Racialized Canadians often face barriers accessing mental health support [59], and we observed a significant effect of racial background on access to 1Call1Click.ca. Clients who identified multiple racial backgrounds (multiracial) or selected “Other” were all represented in the 1Call1Click.ca clientele in higher proportions than the regional population. Indeed, the over-representation of multiracial individuals is the largest effect observed in any of our analyses.

It is worth noting here that clients in the Multiracial grouping were those who indicated two or more racial backgrounds. To the authors’ knowledge, Statistics Canada has not released information on racial background tied to age. However, they have reported that the proportion of people in Canada identifying with multiple races is increasing, particularly among those who are second or third generation immigrants [60]. This may be the explanation for the relatively high proportion of multiracial youth in the client population relative the overall population.

We observed small but significant under-representations of clients identifying as Filipino, Japanese, Chinese, South Asian, and Indigenous. We note that between 18 and 77% of the people who indicated these under-represented identities also endorsed a second identity and were thus counted in the ‘multiracial’ group instead. Other studies in Ontario have observed that both immigrant and Canadian-born Chinese and South Asian individuals were less likely to seek care for MHASUH challenges than White or Black individuals, despite having similar levels of self-reported MHASUH challenges [61].

### Family income

Clients from both the lowest and highest income groups were represented in the 1Call1Click.ca clientele in higher proportions than present in the regional population. We observed no effect of family income on the assigned *Level of Need*, and a small but significant inverse relationship between family income and HEADS-ED total score, wherein the HEADS-ED totals decreased as family income increased. Studies typically show an increase in the incidence of MHASUH challenges and a decrease in self-reported mental health as income decreases, however greater need is not strictly related to greater service utilization [62–64]. One Canadian study found higher levels of service utilization in those with higher income and the lowest levels of service use for those in the middle of the income distribution, similar to the results presented here [63].

### Strengths & limitations

#### Limitations

In using Census data, we are limited to the 2021 data that provided by Statistics Canada. As a result, the data collected for the most recent census required that we make some assumptions to match the dataset collected at 1Call1Click.ca. In cases in which the income bands did not cleanly align between the census and 1Call1Click.ca, for instance, we assumed an even distribution of people across the census distribution to split and match.

It is possible that the decision to treat responses of “I don’t know” or “Prefer not to answer” as missing data, along with the use of pairwise deletion may have had an impact on the outcomes of our analyses. That is, the individuals who could not or did not want to share certain information might differ systematically from those who were willing to or could. Given that the data discussed here is gathered during a healthcare intake (during which the gathering of SDH related information is a secondary goal), our decision to analyze data this way came from the objective of comparison to census data (which does not include “I don’t know” or “Prefer not to Answer” responses) and to include as many of the responses from clients as possible into our analyses. This creates the possibility of difference between the analysis groups, but as our goal was to be as representative as possible, we prioritized inclusion with the belief that doing so would increase the overall representativeness of the sample and of the community. With this in mind, it is important to note that interpretation of the income related results should be limited, due to the small but statistically significant differences between the acuity measures of those who either did or did not provide income data.

We report differences in outcomes for the clinical screeners (HEADS-ED, HEADS-ED Under 6, ASQ, and CRAFFT 2.1 + N). It is possible that the SDH characteristics we discuss in this paper influence the way that clients interpret or respond to the questions in those questionnaires in a systematic way that reflects the enculturation and social expectations of those clients rather than differences in acuity. That said, the screening tools in use at 1Call1Click.ca have been translated into multiple languages and have been used in countries across the world [65–70]. We operate under the assumption that clients, who made the decision to reach out for assistance, are answering intake questions truthfully. However, we cannot rule out that linguistic and cultural differences in willingness to discuss certain topics or the way that the meaning of a question is understood might contribute to these differences.

Clients’ parents and caregivers are often on the intake call with the children and youth contacting the service or may be making the call on behalf of their young children (though calls regarding those aged 12 or higher require the presence of the child/youth on the call). The presence of a parent may additionally enforce the effects of enculturation speculated at above or otherwise impact the nature of the clinical or demographic information provided on the call. The dataset used for these analyses does not include information about the presence of a parent on the call.

There is no baseline dataset for the SDH demographics of child and youth MHASUH needs against which to compare outcomes at 1Call1Click.ca. This limits the extent to which we can interpret the effectiveness of the 1Call1Click.ca program as a mechanism for connecting children, youth and families to care. However, the data and analyses presented here represent an attempt to overcome this limitation. Similarly, the need to collapse certain groups for some of our analyses may obscure some important differences between the experiences of those groups. We hope to examine these experiences and potential intersectional effects in future work.

1Call1Click.ca is a close approximation but not a direct measurement of access to MHASUH care. While 1Call1Click.ca does connect each client who goes through an intake with one or more services and provide waitlist supports and information, it does not directly provide counselling or therapy services. We cannot presently speak to follow-through, referral outcomes, or length of time on a waitlist, though these will be the subject of future work.

#### Strengths

This study highlights the strength of 1Call1Click.ca towards the accessibility of MHASUH care, one of the primary goals of the service. While we cannot compare to data from before the implementation of 1Call1Click.ca, the data we do have indicate that in most respects that goal is being achieved.

We performed these analyses using real world health data collected by intake workers at a live service. The Census is another real-world dataset that is publicly available and free to access. These types of analyses are therefore reproducible to any Canadian service (or service in any country with high quality and publicly available census data) that wishes to evaluate their accessibility in the absence of historical or baseline data.

Comparing client proportions to population proportions is a useful and informative method for services to use to evaluate accessibility. Moreover, this data allows the service to monitor and respond to new challenges, challenges that under most previous circumstances would not have been quantifiable. For instance, the service can engage in targeted outreach to groups that are under-represented amongst their clientele and then monitor if that outreach leads to improvements. Importantly, the “baseline” of data that we lacked initially now exists. This data will be a great benefit for future research and healthcare service planning.

### Implications and future directions

These analyses create the opportunity for future targeted outreach to groups who are not accessing services proportionally and investigation of barriers to under-represented groups. Specifically, cisgender girls under 10 and cisgender adolescent boys, moderate income families, and people from under-represented racial backgrounds are groups that could be targeted for outreach. Moreover, service providers will be able to consider SDH and demographic factors in service planning at a local level and, where appropriate, implement targeted and culturally appropriate services.

One outcome of interest was the size of the “multiracial” group in our analyses, which was composed of individuals identifying themselves with more than one racial background. The changing demographics in Canada and the results we observed suggest that this is an area where future research would be useful, to understand the unique needs of this growing population more clearly [60].

Longitudinal studies of 1Call1Click.ca access patterns over time will be possible, allowing evaluation of the effectiveness of any attempts at targeted outreach as well as follow-up on variables including rates of program completion and clients’ MHAUSH outcomes. Likewise, as the dataset grows, we may be able to conduct more fine-grained analyses to examine potential intersectional effects.

These data and analyses will be particularly useful in future census years to track progress, or to measure the impact of future changes to the local MHASUH landscape due to changes in funding, new programs, legislation, or major public health issues (such as a future pandemic). We are also specifically interested in the patterns of access for specific age groups, particularly infants and children under 6 years of age.

## Conclusions

Clients endorsing characteristics that have historically faced disproportionate barriers in accessing care mostly accessed 1Call1Click.ca in proportions representative of or exceeding those of the regional population. Specifically, cisgender female, non-binary, and transgender clients were present in higher-than-population proportions, as were clients of the multiracial and “other” racial background, and clients at the lowest ($0–29,999) and highest ($150,000 or more) annual family income levels. Cisgender-male clients, Filipino, Japanese, Chinese, South Asian, and Indigenous clients, and those reporting family income of $60,000 to $89,999 were under-represented relative to census data. We observed only small effects for those appearing in lower-than-population frequencies. All other groups were present at community levels.

We observed either no or small effects of SDH characteristics on the acuity of clients MHASUH needs at presentation to 1Call1Click.ca. For gender, we saw a very small difference in average HEADS-ED total score in which cisgender male clients had higher scores than cisgender female clients. We also observed higher risk outcomes on the ASQ for cisgender female, transgender, and non-binary clients relative to cis-gender males. For income, this included a small effect where higher income levels had lower HEADS-ED totals, and a small difference in the HEADS-ED Under 6 total score between the highest and second lowest income groups. We observed small effects wherein White clients were assessed at higher CRAFFT and Level of Need, where racialized clients were at higher risk on the ASQ. There were no characteristics for which differences in acuity were detected by all measures used.

These results combined suggest that 1Call1Click.ca has largely been effective in being accessible to children and youth from groups who have historically faced disproportionate barriers in accessing care. 1Call1Click.ca was created with the acknowledgement that structural barriers exist, are not randomly or evenly distributed, and shape both who is able to access care and how difficult it is to do so. This indicates the potential of regional coordinated access and service navigation programs in facilitating access to child and youth MHASUH care and addressing the inequities related to social determinants of health.

## Supplementary Information

Supplementary Material 1.

## Data Availability

The dataset analysed in this manuscript is not and will not be made publicly available. It contains private health information which, even in the absence of direct identifiers, could be potentially identifying in combination. For questions about the data, please contact Scott Robson at srobson@cheo.on.ca.

## Abbreviations

ANOVA: Analysis of Variance
ASQ: Ask Suicide Questions- A screening tool used to assess suicidality
CRAFFT: A youth substance-use screening tool, an acronym of Car, Relax, Alone, Forget, Family/Friends, Trouble
HEADS-ED: A general mental health and addictions screening tool for child and youth aged 6 and older. A mnemonic from the domains it assesses: Home, Education, employment, Activities and peers, Drugs and alcohol, Suicidality, Emotions, behaviours, thought disturbance, and Discharge or current resources
HEADS-ED Under 6: A general mental health for infants and children under the age of 6. A mnemonic of the domains the tool is used to assess: Home and caregivers, Eating and sleeping, Activities and peers, Development, speech/language/motor, Safety, Emotions, behaviours, and Discharge or current resources
MHASUH: Mental Health, Addictions, and Substance Use Health
OR: Odds Ratio
STROBE: Strengthening the Reporting of Observational Studies in Epidemiology- guidelines for reporting observational studies
