# Supplementary material for: Equity and access to a regional child and youth mental health and addiction navigation service: a secondary analysis study
Source: BMC Public Health. 2026 Jun 6;26:2324. doi: 10.1186/s12889-026-28043-3 (PMC13459356; doi:10.1186/s12889-026-28043-3)
Supplement: Supplementary file 1 [file 12889_2026_28043_MOESM1_ESM.docx]

Appendices

Appendix A

*Gender*

Client’s information includes their sex at birth and their self-identified gender identity. “Sex at birth” information was available for 9414 clients while “gender identity” information was available for 3405. We combined these two variables, prioritizing the stated gender identity information where available and using the “sex at birth” information where gender identity was not available. This provided a value for 9454 (97.5%) clients, of whom 9370 were included in the analyses (96.6%). To align with the census categorization, client responses of “transgender male” (n=67) and “transgender female” (n=10), as well as individuals whose stated gender identity of “male” (n=56) or “female” (n=18) did not match their sex at birth, were added into the category “transgender” (n=151). Analyses included the categories of cisgender-female, cisgender-male, non-binary, and transgender. Because the CRAFFT 2.1+N is used with fewer clients, the categories of non-binary and transgender are combined for those analyses. Other responses (Table 1) were not included due to either a small number of clients endorsing that identity or a lack of information on that identity within the Census results.

*Income*

Family income information was available for 3860 clients (39.8%) and included 6 ranges of annual family income (see Table 1). All incomes are described in Canadian Dollars (CAD). Over 50% of clients indicated that they either did not know (n=4022) or would prefer not to answer (n=814) and these clients were not included in income analyses.

*Racial Background*

Racial background information was provided by 9179 (94.6%) of clients and included 14 racial backgrounds (see Table 1). Clients for whom no data was recorded (n=379) or who selected “Prefer not to answer” (n=136) were not included in these analyses. The category “Multiracial” was not an option available to clients but rather is a constructed code that encompasses all clients who indicated more than one racial background. Due to the group sizes, the *Level of Need*, CRAFFT 2.1+N, ASQ, HEADS-ED, and HEADS-ED Under 6 analyses used a constructed variable (“Racialized”, n=2248) which includes all individuals endorsing racial backgrounds other than “White”.

*Clinical Information*

The HEADS-ED was used to screen all 8970 (92.5%) clients who were between the ages of 6 and 21. The HEADS-ED Under 6 was used to screen all 724 (7.5%) clients who presented prior to their 6^th^ birthday. The CRAFFT 2.1+N was administered to 4171 (43.0%) clients who presented after their 12^th^ birthday. 3744 (38.6%) clients were screened using the ASQ. The A *Level of Need* was assigned to 8982 (92.7%) clients. See Table 1 for a description of each *Level of Need* and the distribution of clients across the levels.

Appendix B

Matching to Census

*Income*. 1Call1Click.ca provides six income bands for clients to choose from, where the census included 19. The census bands were combined to match; for instance, the respondents to the census options of “$35,000 to $39,999”, “$40,000 to $44,999”, “$45,000 to $49,999”, and “$50,000 to $59,999” were combined to match the 1Call1Click.ca income band “$30,000 to $59,999”. The boundaries between these typically lined up, though in one instance they did not. The census includes “$100,000 to $124,999” while 1Call1Click.ca includes “$90,000 to $119,999” and “$120,000 to $149,999”. In this instance, an even distribution was assumed across the census group and the persons within that group were allocated proportionately to the appropriate group (80% to “$90,000 to $119,999” and 20% to “$120,000 to $149,999”).

*Gender Identity.* The census provides gender data for “Male +” and “Female +”, which includes transgender and cisgender people in the gender category they identify with. However, Statistics Canada provided separate data on the proportions of transgender and non-binary people in the 1Call1Click region.^54^ For the purpose of comparison to the 1Call1Click.ca data, the provided proportions of transgender and non-binary people were subtracted from the census numbers provided for “Male +” and “Female +”.

*Racial Background*. The census included an option for individuals to indicate that they identify with multiple racial backgrounds (“multiple visible minorities”), whereas 1Call1Click.ca does not, instead allowing clients to make multiple selections. For our analyses, those who selected multiple backgrounds were combined into a single category “Multiracial”, and the size of this group was compared to the “multiple visible minorities” response on the census. Additionally, the census category “Not a visible minority” includes both people who identify as “White” and those who identify as “Indigenous”. For the purposes of matching the 1Call1Click.ca responses of “White” and “Indigenous”, the number of individuals on the census who indicated “Indigenous ancestry (only)” were subtracted from the “Not a visible minority” count and used as its own category (“Indigenous”) and the remainder were compared to the “White” respondents.
